# Supplementary figures and images for: Increased resting heart rate indicates high-workload hearts with augmented aortic hydraulic power in hypertensive pigs
Source: PLoS One. 2025 Jan 13;20(1):e0316607. doi: 10.1371/journal.pone.0316607 (PMC11729957; doi:10.1371/journal.pone.0316607)

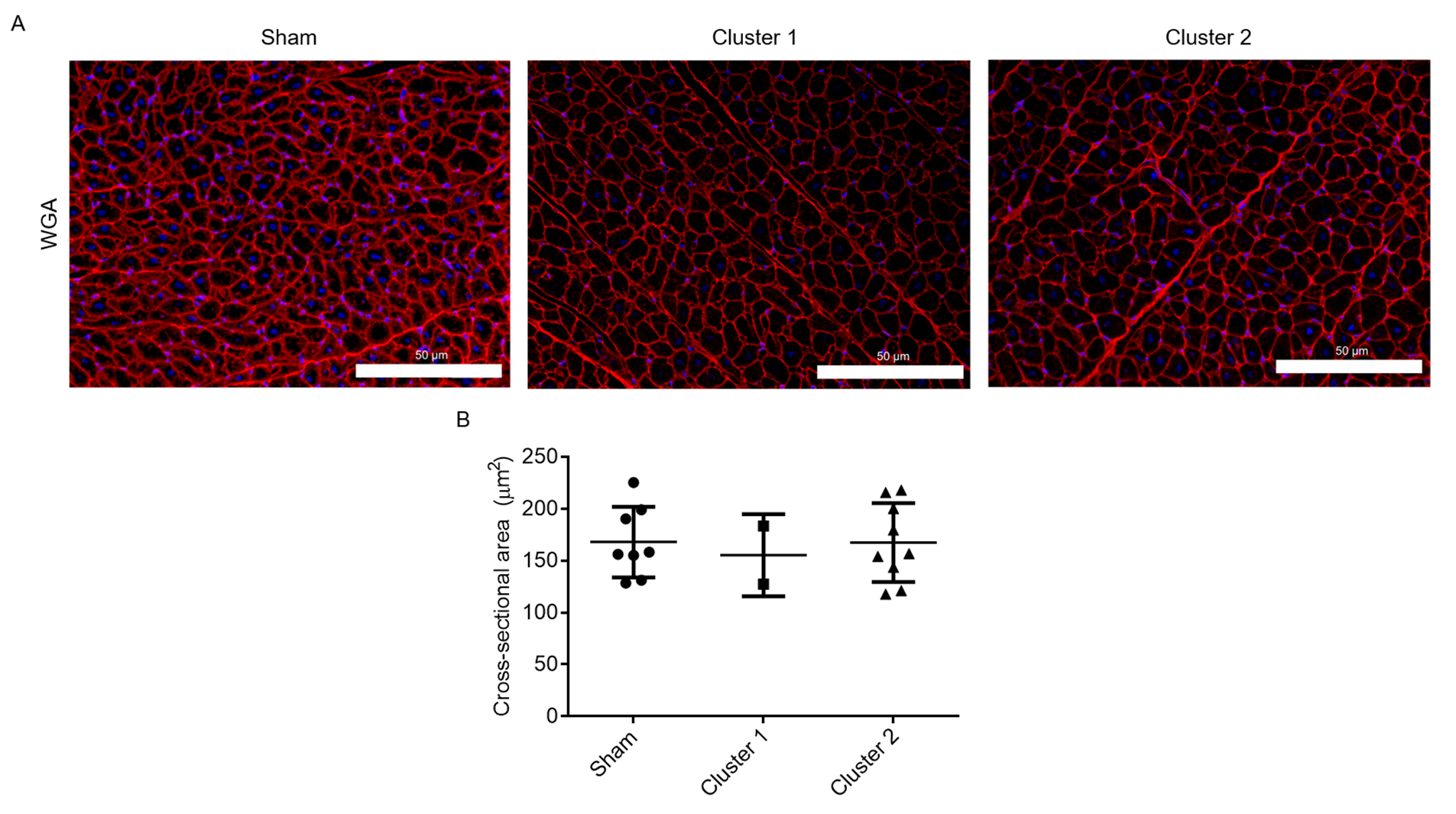

Supplement: S1 Fig — The cross-sectional area of cardiomyocytes was assessed with fluorescent images of wheat germ agglutinin (WGA)- and Hoechst 33342-stained cells. Sham group, n = 8 (2F, 6M); Cluster 1 group, n = 2 (1F, 1M); Cluster 2 group, n = 9 (3F, 6M). Data are expressed as mean ± SD. F: female, M: male. (TIF) [file pone.0316607.s001.tif]

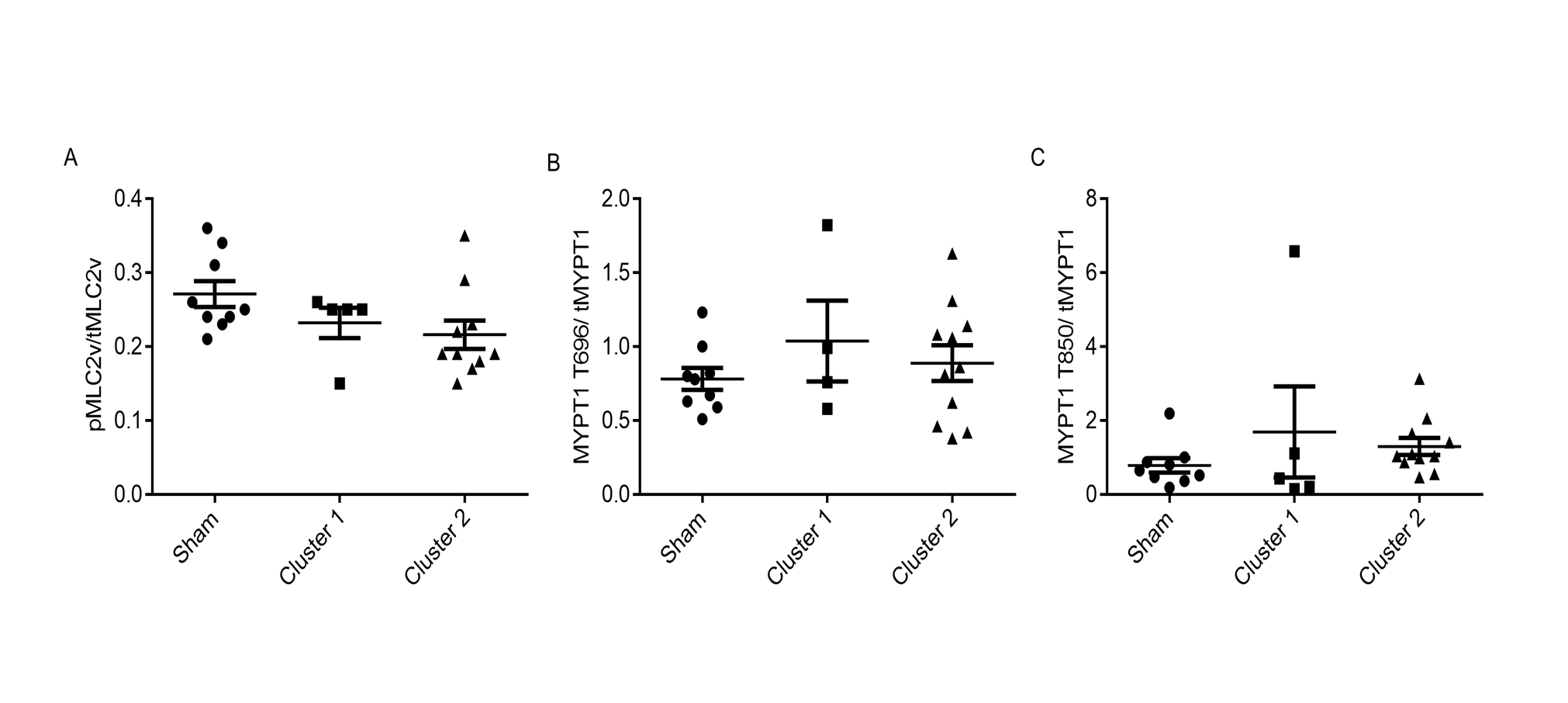

Supplement: S2 Fig — Activation levels of two cardiac hypertrophy biomarkers, MLC2v and ROCK 1/2, were examined in the left ventricle (LV) of sham, Cluster 1, and Cluster 2 groups. MLC2v activation was assessed with Phos-tag SDS-PAGE with subsequent immunoblotting to detect the phosphorylated and non-phosphorylated MLC2v (A). Sham group, n = 9 (3F, 6M); Cluster 1 group, n = 5 (2F, 3M); Cluster 2 group, n = 10 (3F, 7M). ROCK 1/2 activation was assessed with the phosphorylation levels (T696 and T850) of myosin phosphatase target subunit 1 (MYPT1, B). Sham group, n = 9 (3F, 6M); Cluster 1 group, n = 4 (2F, 2M) for T696, n = 5 (2F, 3M) for T850; Cluster 2 group, n = 11 (3F, 8M). Data are expressed as mean ± SD. F: female, M: male. (TIF) [file pone.0316607.s002.tif]
