# Supplementary material for: Increased resting heart rate indicates high-workload hearts with augmented aortic hydraulic power in hypertensive pigs
Source: PLoS One. 2025 Jan 13;20(1):e0316607. doi: 10.1371/journal.pone.0316607 (PMC11729957; doi:10.1371/journal.pone.0316607)

Fig5_A_Sham

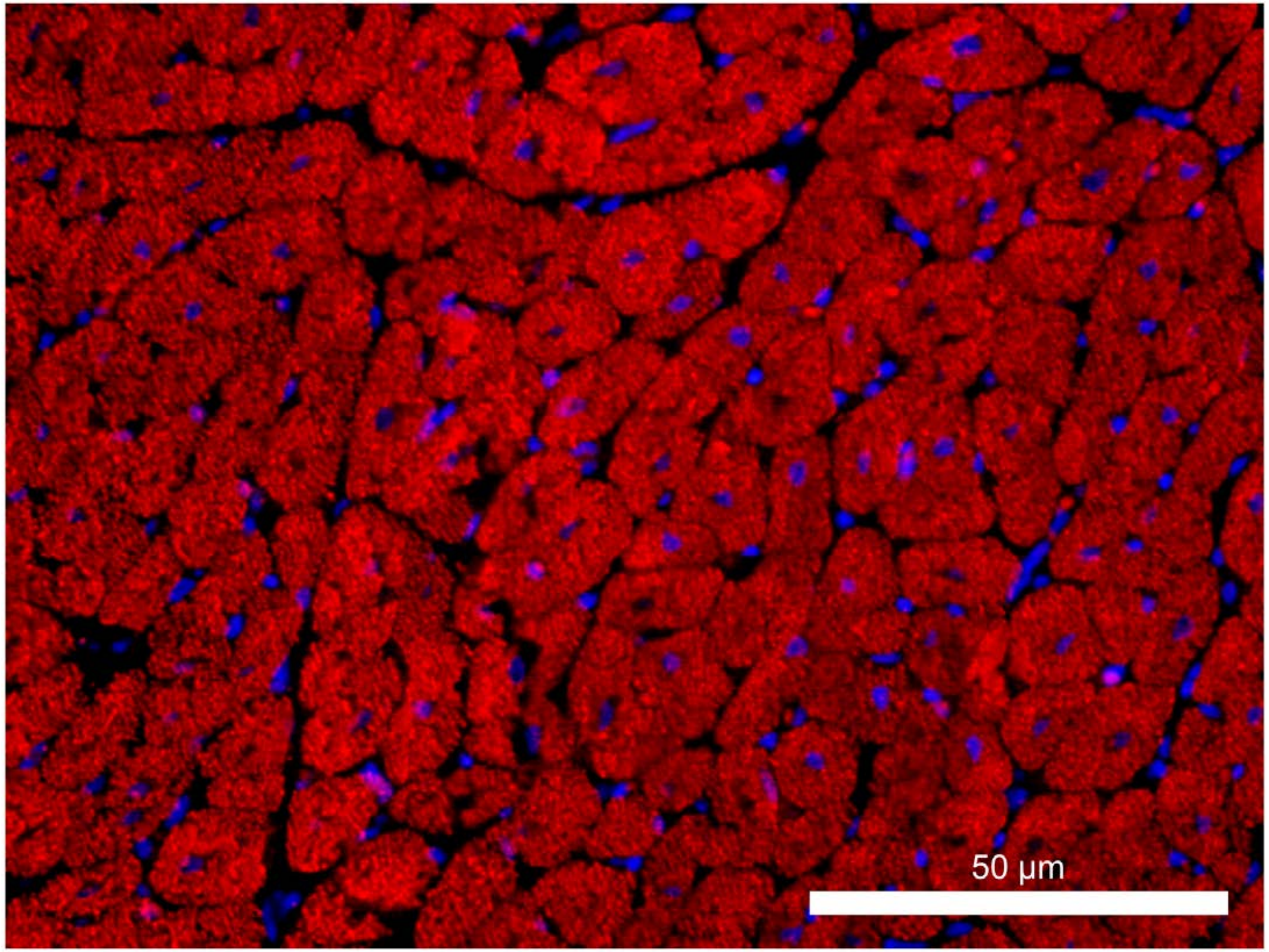

Fig5_A_Cluster 1

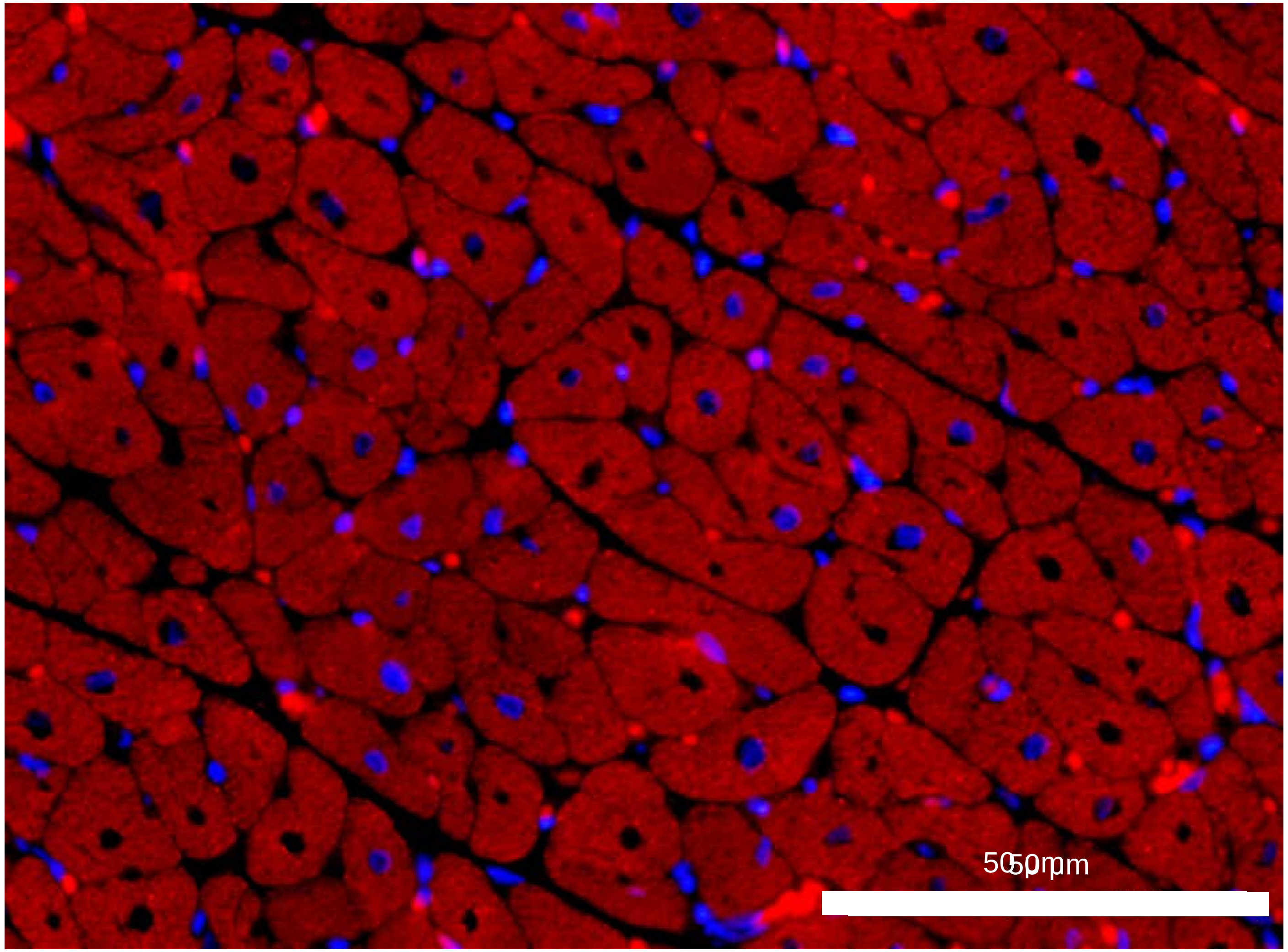

Fig5_A_Cluster 2

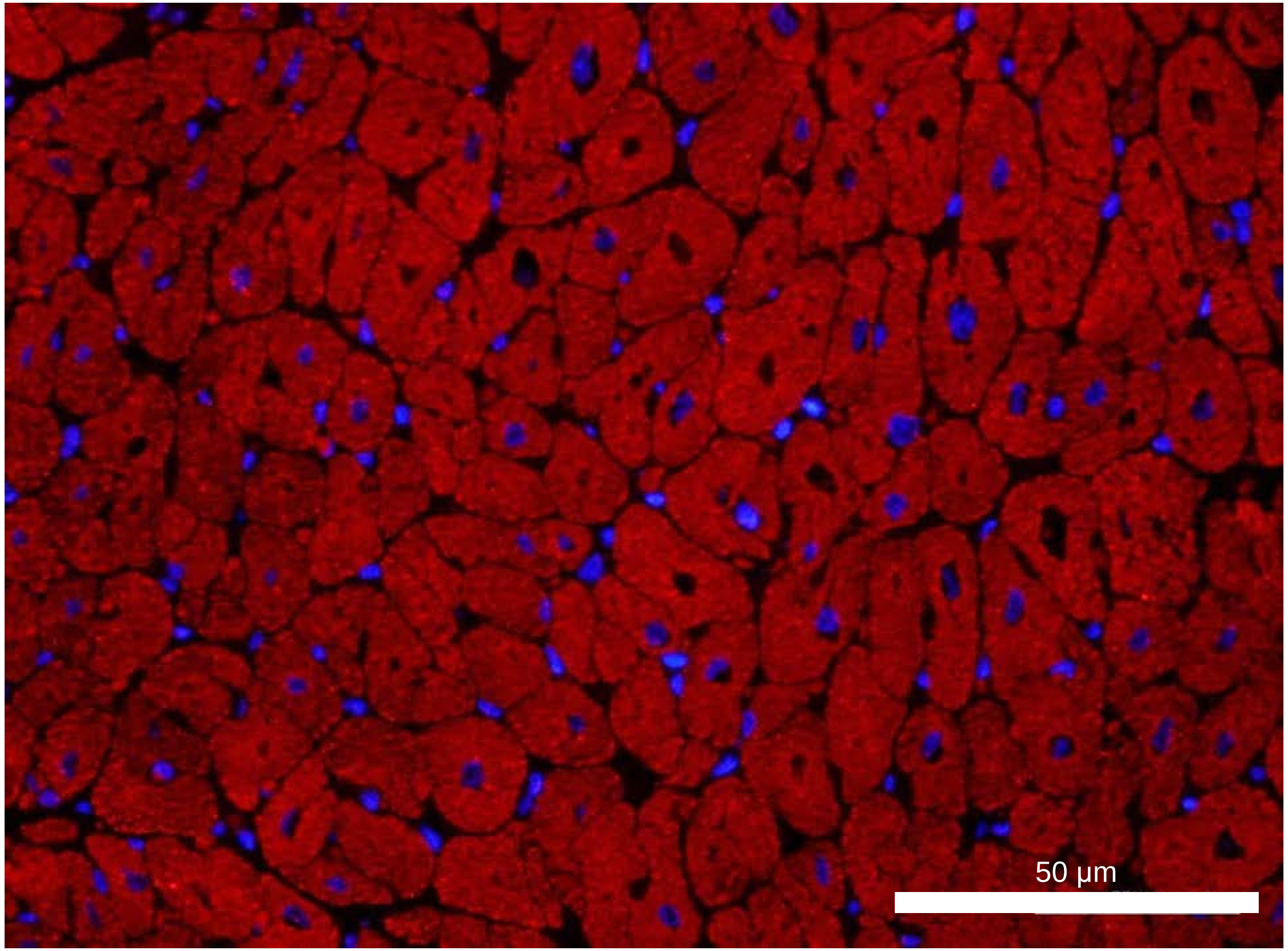

Fig5_B_ Sham

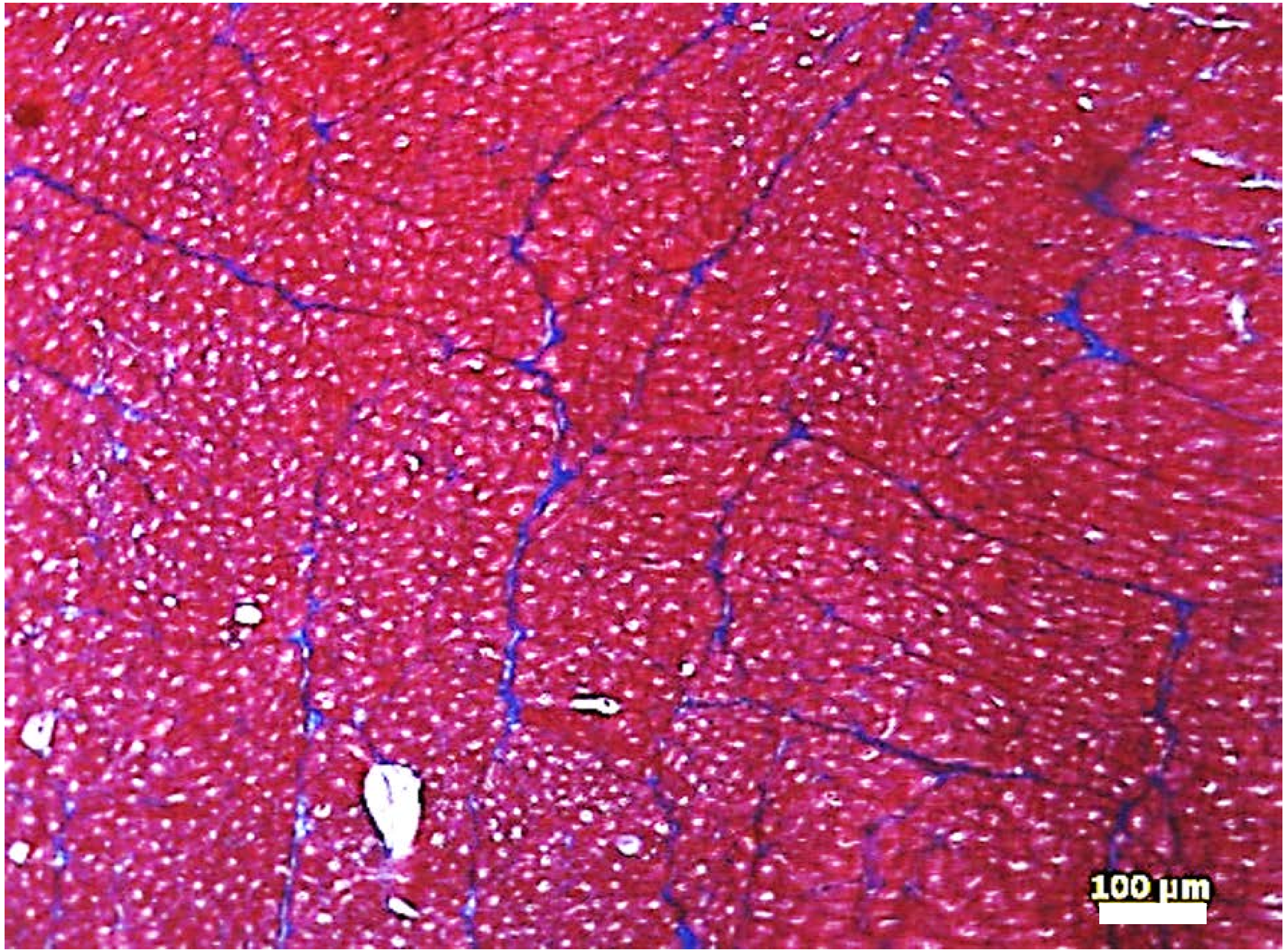

Fig5_B_Cluster 1

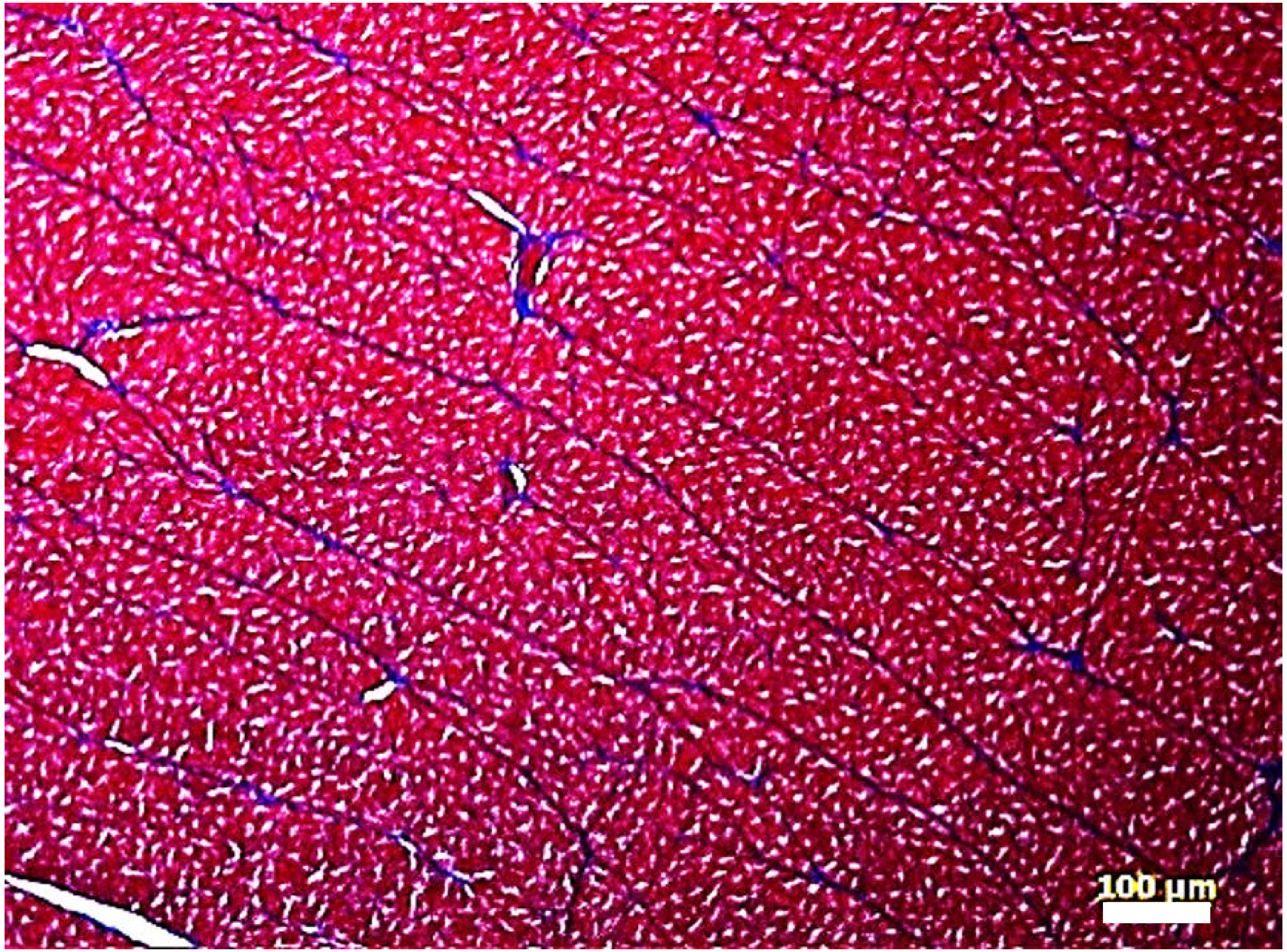

Fig5_B_Cluster 2

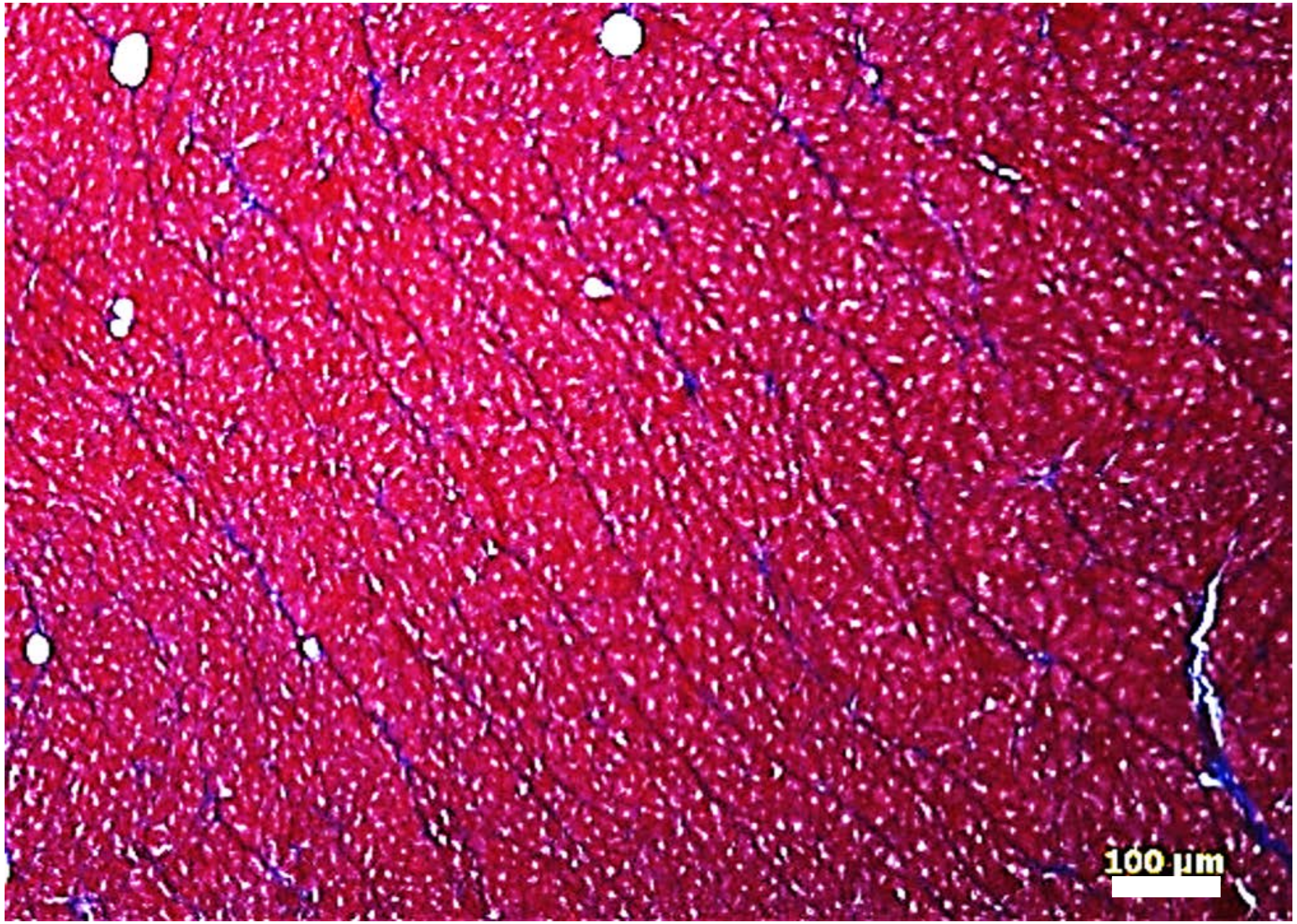

S1_Fig Sham

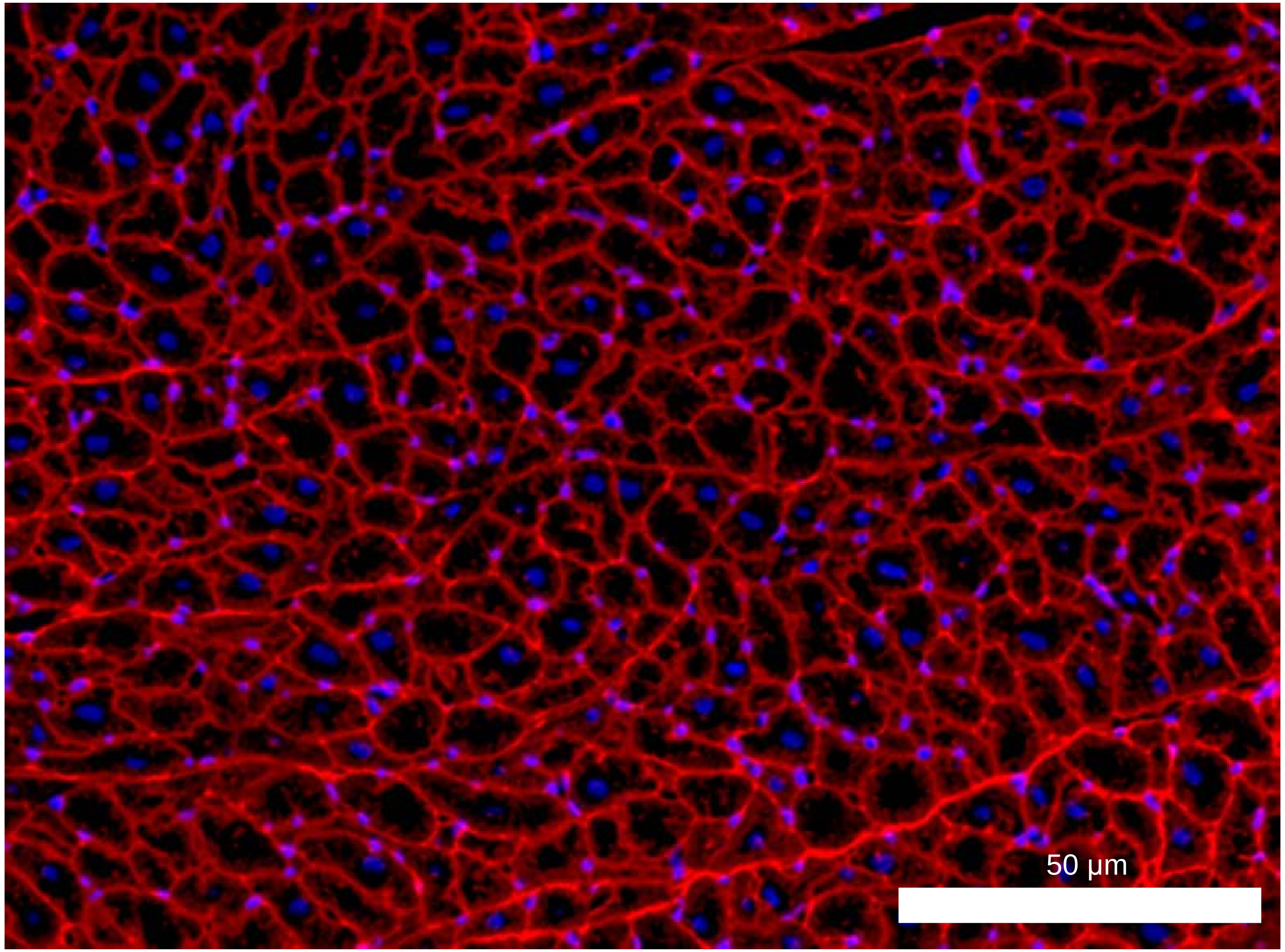

S1_Fig Cluster 1

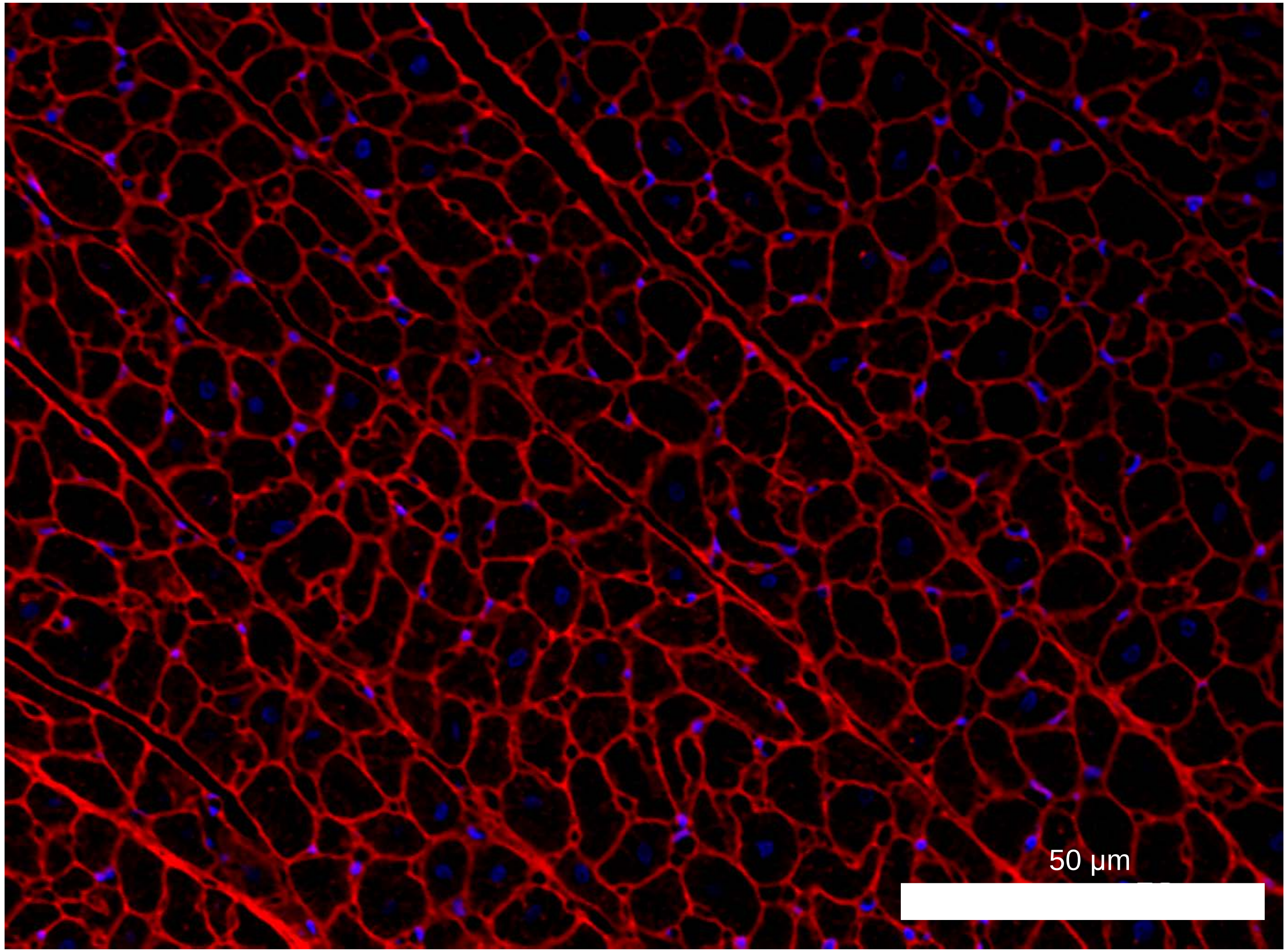

S1_Fig Cluster 2

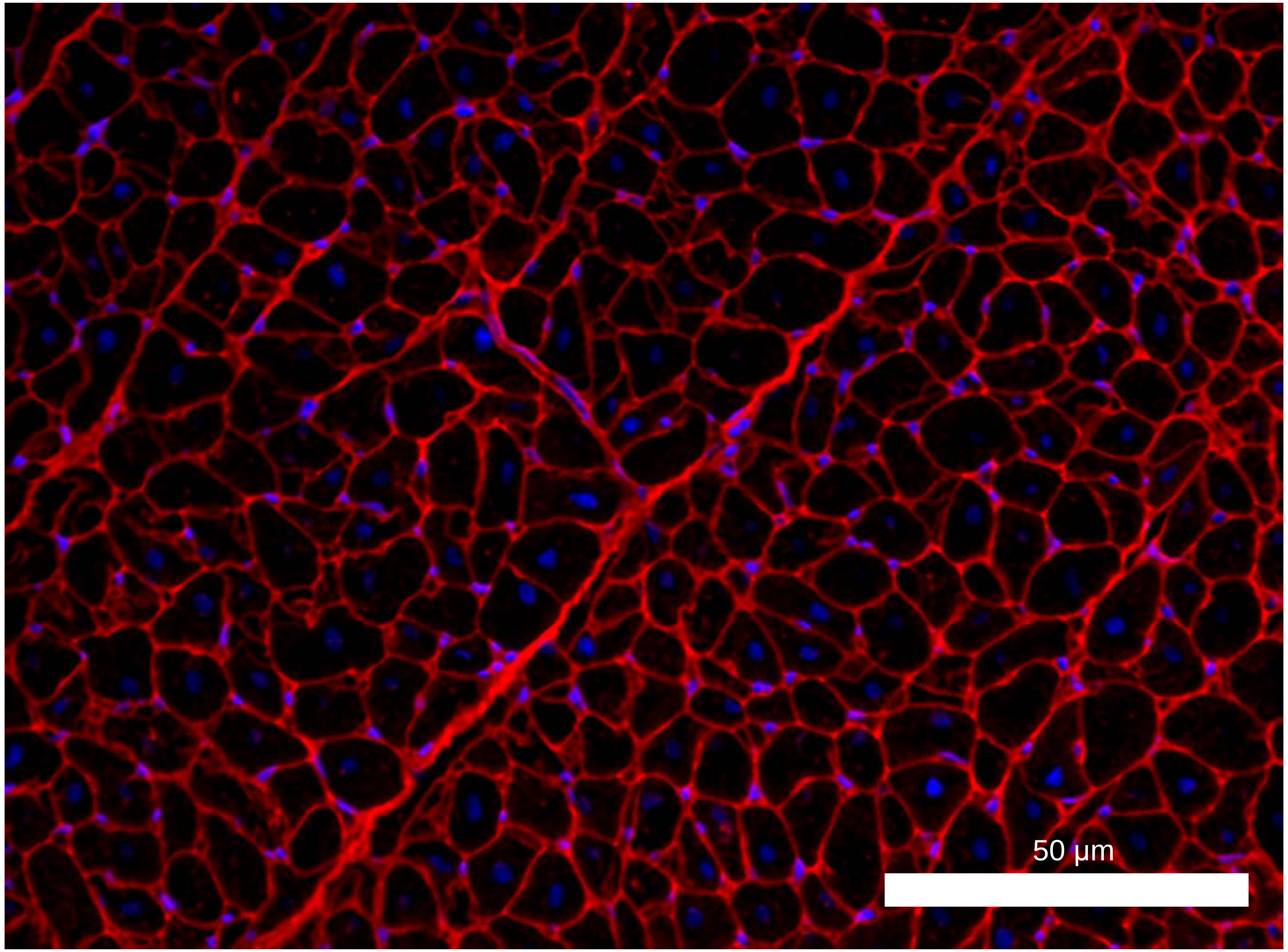

Fig6_A_HSP90

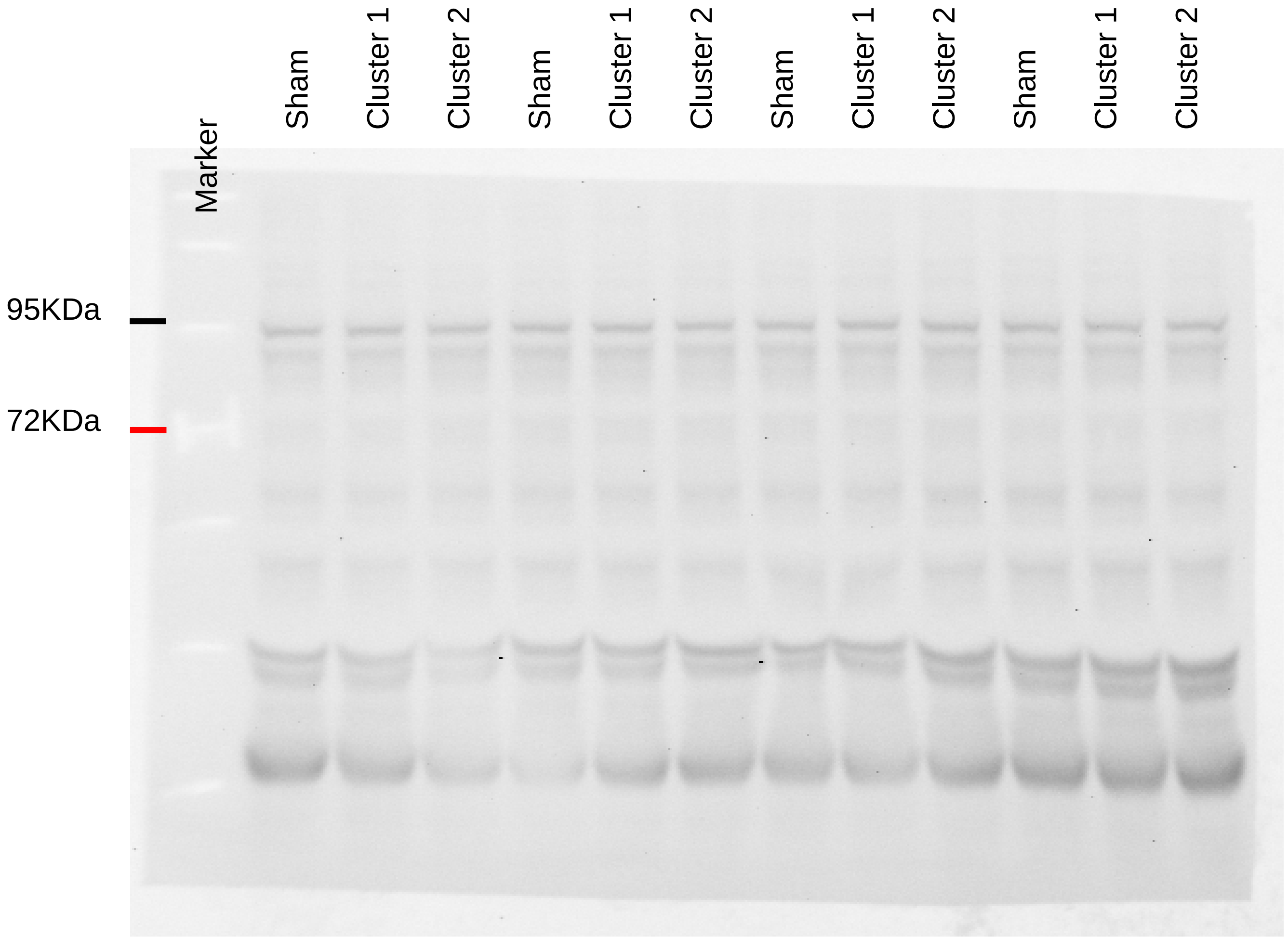
Marker
Sham
Cluster 1
Cluster 2
Sham
Cluster 1
Cluster 2
Sham
Cluster 1
Cluster 2
Sham
Cluster 1
Cluster 2
95KDa
72KDa

Fig6_A_ HSP70

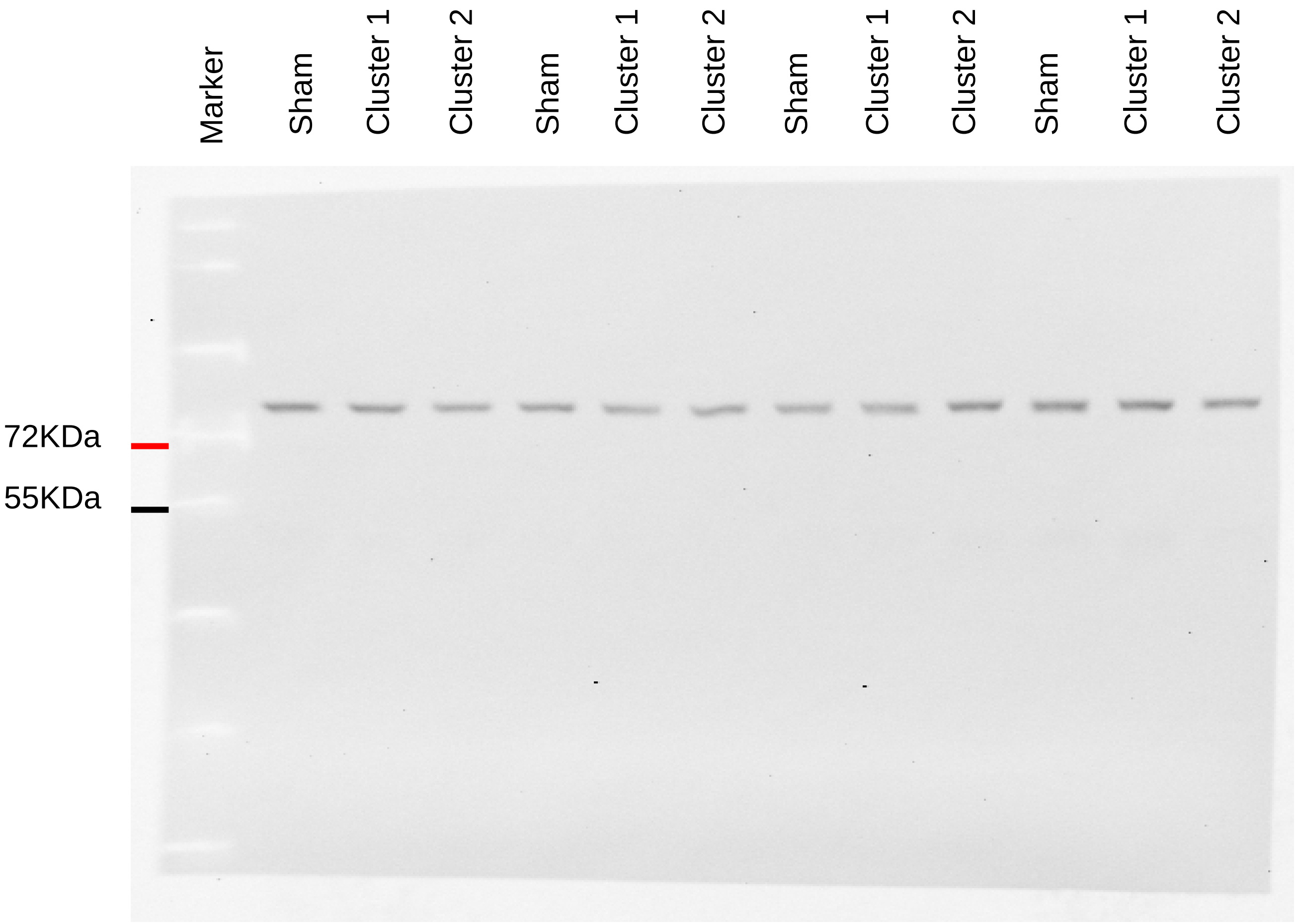
Marker
Sham
Cluster 1
Cluster 2
Sham
Cluster 1
Cluster 2
Sham
Cluster 1
Cluster 2
Sham
Cluster 1
Cluster 2
72KDa
55KDa

Fig6_A_HSP60

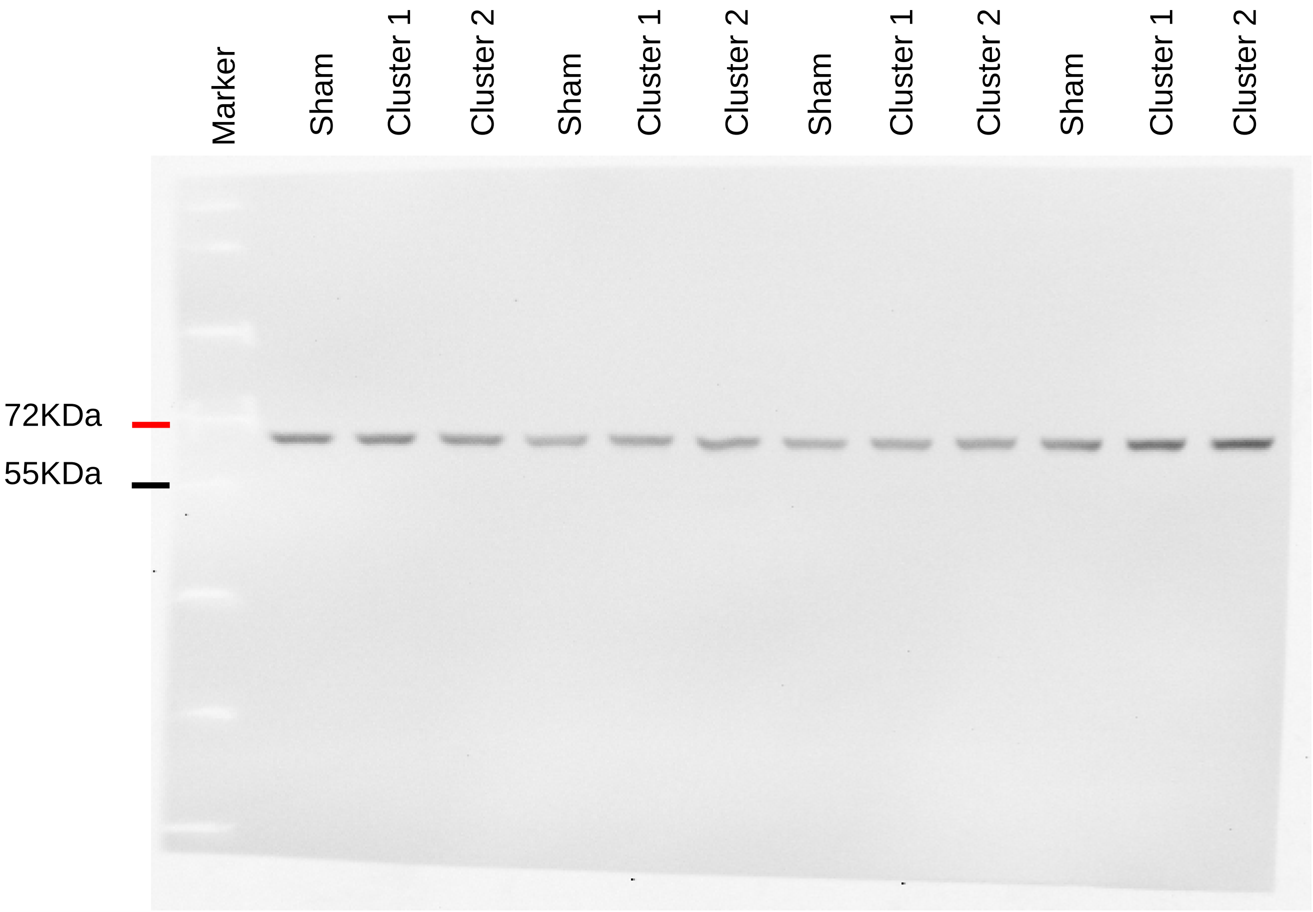

Marker
Sham
Cluster 1
Cluster 2
Sham
Cluster 1
Cluster 2
Sham
Cluster 1
Cluster 2
Sham
Cluster 1
Cluster 2
72KDa
55KDa

Fig6_A_HSP40

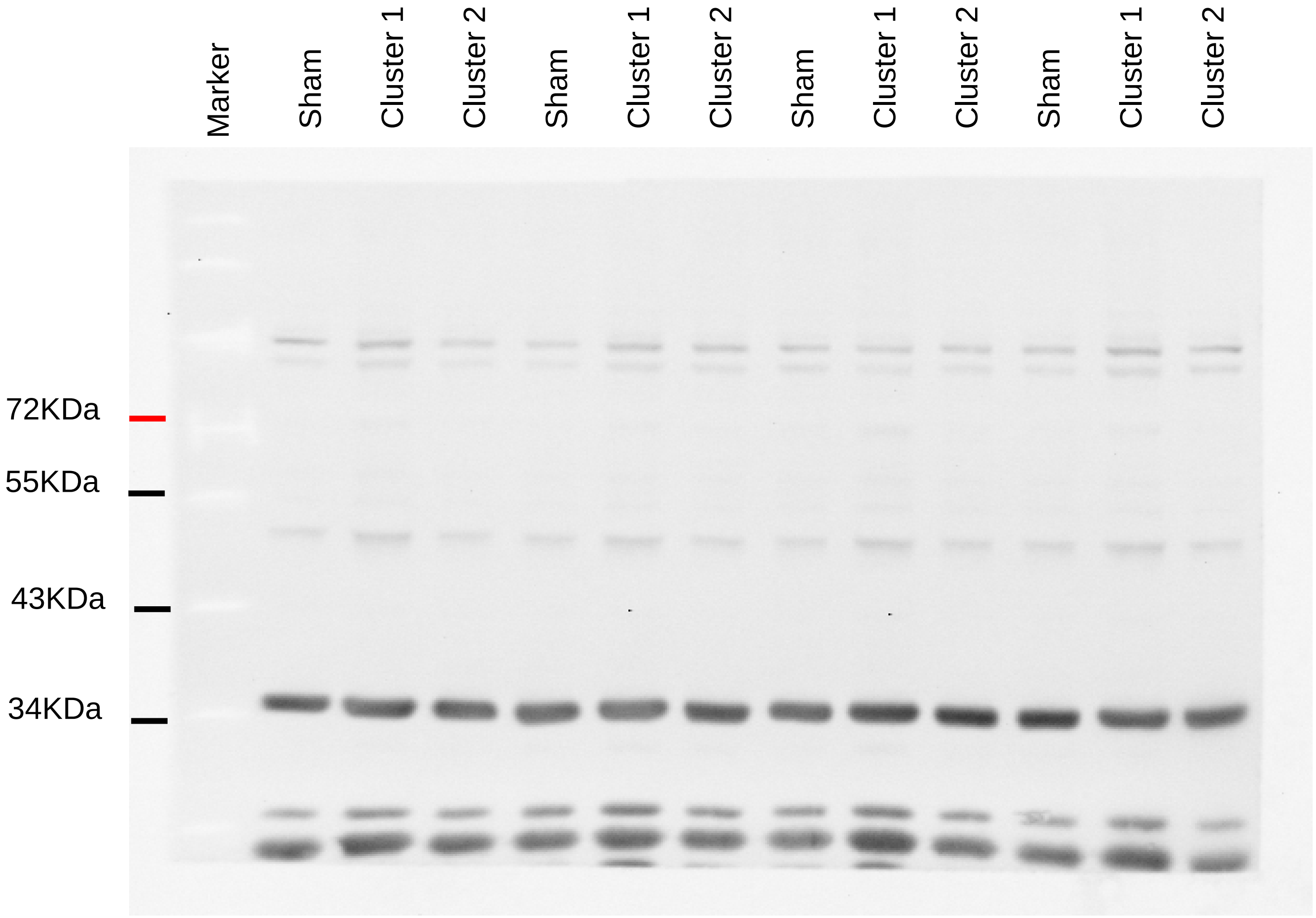
Marker
Sham
Cluster 1
Cluster 2
Sham
Cluster 1
Cluster 2
Sham
Cluster 1
Cluster 2
Sham
Cluster 1
Cluster 2
72KDa
55KDa
43KDa
34KDa

Fig6_A_α-tubulin (HSP)

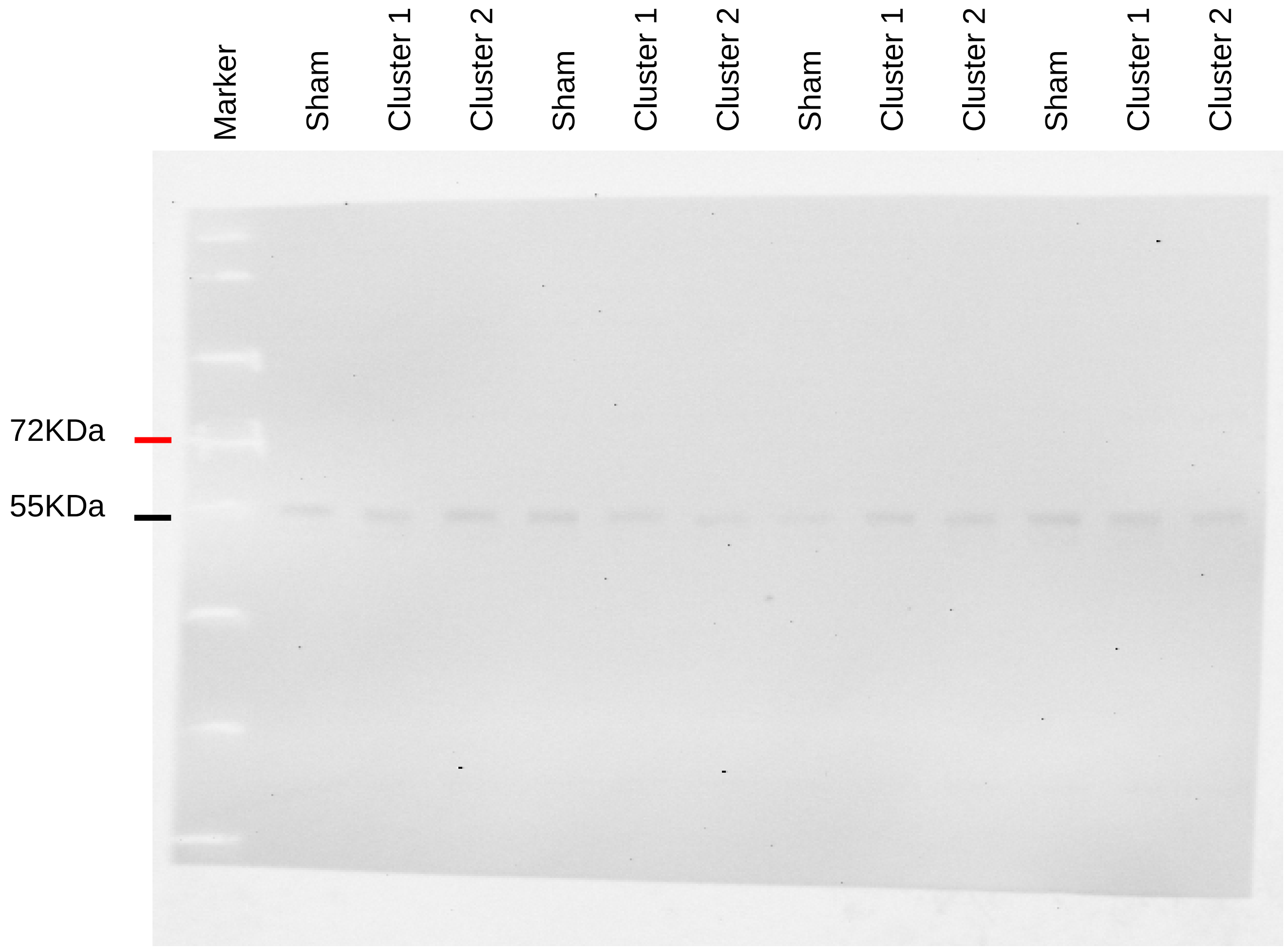

Fig6_A_Osteopontin

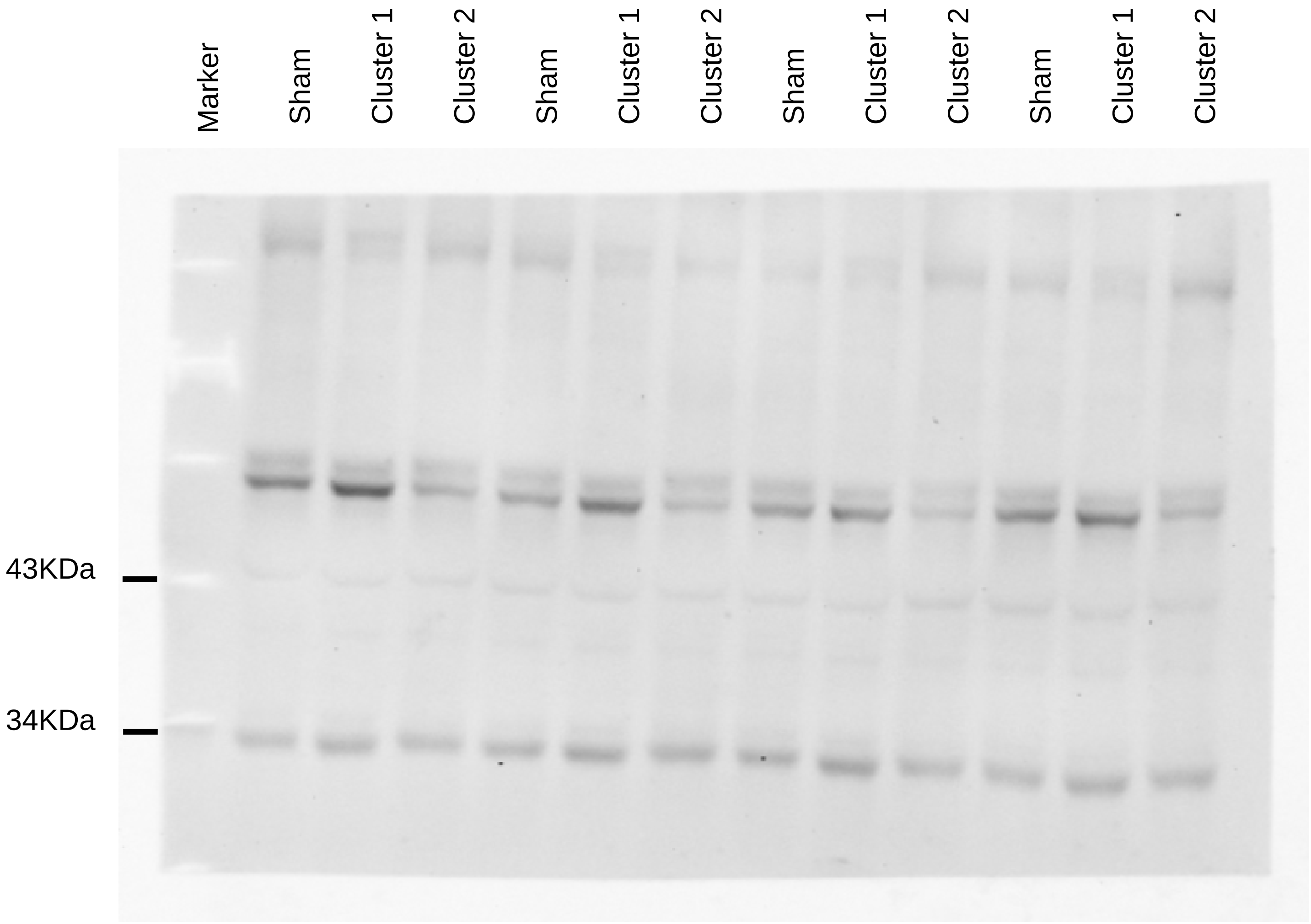

Fig6_A_α-tubulin (Osteopontin)

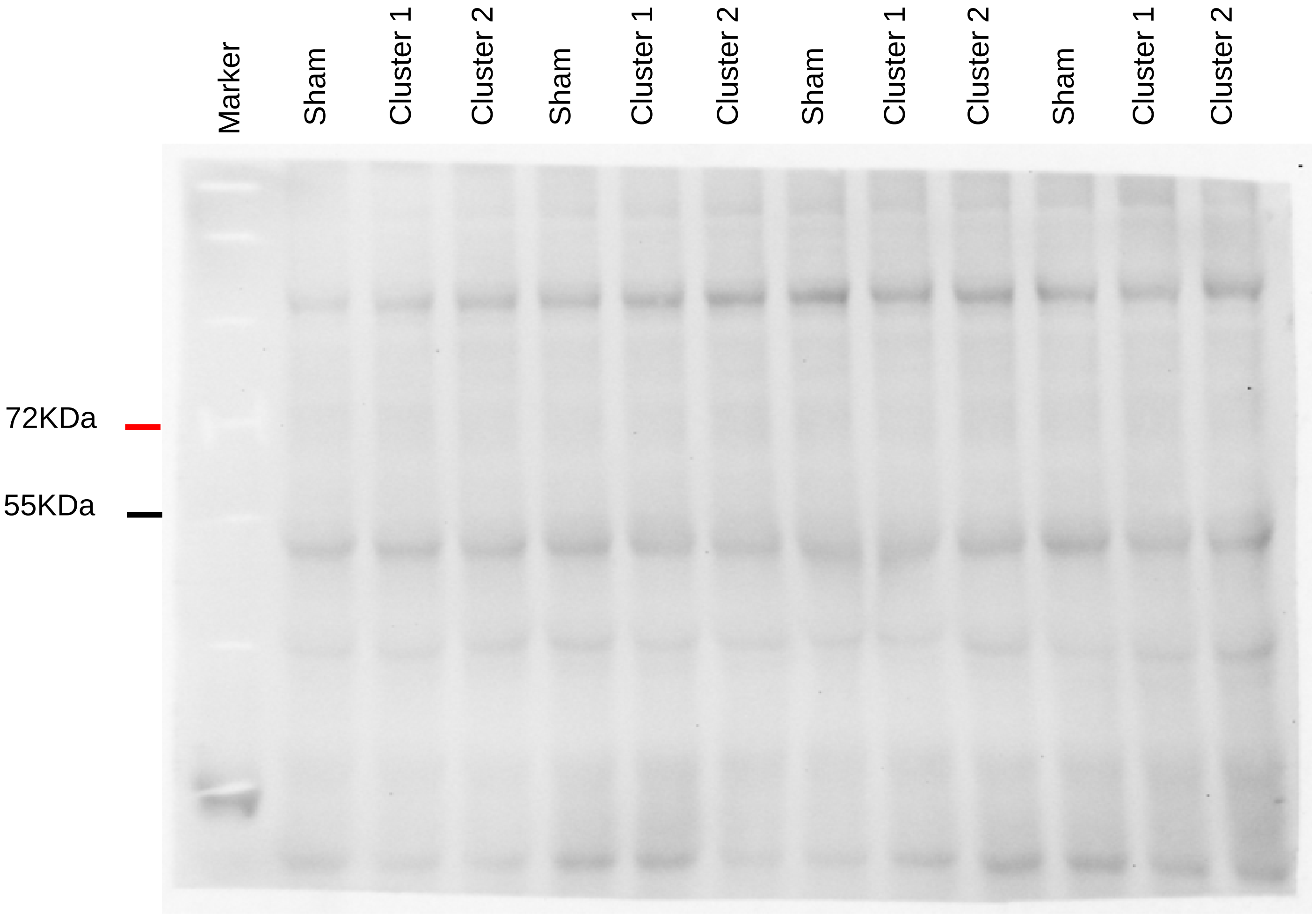

Supplement: S1 Raw images — (PDF) [file pone.0316607.s004.pdf]
