# Supplementary material for: Increased resting heart rate indicates high-workload hearts with augmented aortic hydraulic power in hypertensive pigs
Source: PLoS One. 2025 Jan 13;20(1):e0316607. doi: 10.1371/journal.pone.0316607 (PMC11729957; doi:10.1371/journal.pone.0316607)
Supplement: Attachment — Submitted filename: Response_to_Reviewers.docx [file pone.0316607.s005.docx]

**Responses to Reviewers**

**Responses to comments of reviewer 1**

**Comment 1:** *The title suggests (incorrectly) that increased heart rate is a cause of augmented aortic hydraulic power. Please rewrite accordingly.*

**Response:**

Aortic hydraulic power, analogous to cardiac power output (CPO), is the product of pressure and volume flow rate (equivalent to cardiac output (CO)) measured at abdominal aorta (AA) proximal to coarctation. Augmented aortic hydraulic power indicates increased CPO to overcome afterload enhancement exerted by hypertension (detected in the carotid artery) and to provide adequate CO. Because AA pressure didn’t increase (Figure 2A), augmentation of aortic hydraulic power was due to increased CO. CO = HR x stroke volume, and stroke volume (represented by aortic hydraulic energy (Figure 2F)) didn’t change in our experimental pigs at sacrifice. Accordingly, CO increase is likely attributed to elevated heart rate (HR). Taken together, our results suggest that elevated resting HR indicates high-workload (strenuous) heart in the early stage of hypertensive heart disease (HHD). To clearly describe the findings of our study, we edited the title as “Increased resting heart rate indicates high-workload hearts with augmented aortic hydraulic power in hypertensive pigs”.

**Comment 2**: *The introduction is broad and hard to follow. Overall, the authors should better highlight the current gaps in the literature and the rationale for the present study.*

**Response:**

Thank you for your suggestion. We revised the introduction to be more concise and focused with all changes marked in red. In the introduction of the revised manuscript, we highlighted the current gaps in investigating the pathophysiologic reactions of the early-stage HHD which is modifiable to prevent the proceeding to heart failure. Therefore, we made an effort to clarify the pathophysiology of early HHD through our porcine model.

**Comment 3**: *Notably, the first aim of the study was to "clarify whether hypertension develops in AA coarctation porcine model". However, the authors stated in lines 113-115 that they already showed the effectiveness of their model in inducing hypertension (PMID: 29615895). In fact, Figure 1 of both papers is very similar (if not identical). Did you use the same animals? Please clarify.*

**Response:**

Thank you for your suggestion and amendment. Our previous publication “Hypertension Accelerates Alzheimer's Disease-Related Pathologies in Pigs and 3xTg Mice” in Front Aging Neurosci. 2018;10:73 addressed that hypertension can be induced by moderate coarctation at infra-renal abdominal aorta in pigs. However, blood pressure was measured by cuff sphygmomanometer which is easily confounded by environmental and physiological stimuli. Therefore, we conducted intra-arterial pressure measurement, the gold standard method, via right carotid artery to verify if hypertension did occur in our porcine model. We have modified study aim 1 in the revised manuscript (**lines 80-81**).

To make the comparison between intra-arterial pressure (ABP) and cuff blood pressure (CBP) methods of blood pressure measurement, Figures 1D-F (CBP) have been changed to scattered distribution with comparison of pre-coarctation and sacrifice similar to Figure 1 A-C (ABP). The pigs included in this study are overlapped with those reported in the previous paper but not the same.

**Comment 4**: *Lines 116-120: What does Alzheimer's disease have to do with this study?*

**Response:**

Thank you for your suggestion. Alzheimer's disease really has no correlation to this study and we removed this sentence in the new version of manuscript.

**Comment 5**: *The time frames for the cluster analysis are not clear. Was it at 8 or 12 weeks? The authors stated that coarctation induced hypertension in all animals at week 12. However, in the cluster analysis, only animals in cluster 1 displayed increases in blood pressure. This is confusing.*

**Response:**

Cluster analysis was performed on pigs underwent AA coarctation, covering all time frames. Cluster analysis was based on the classification of AA coarctation pigs with (cluster 1) or without (cluster 2) augmented aortic hydraulic power, representing increased left ventricular workload or strenuous heart. Through cluster analysis, we found that cluster 1 pigs exhibited marked increases in both carotid arterial pressures and mean heart rate (Figures 3C & 4). Among five cluster 1 pigs, four belonged to the group of 12 weeks post-coarctation. Such a temporal association implies that AA coarctation, if applied long enough, can induce hypertension and result in strenuous heart with elevated aortic hydraulic power. Because our experimental period was set for 12 weeks, longer study period may be required to clarify whether AA coarctation induces hypertension concomitant with augmented aortic hydraulic power in all pigs. This perspective is added in the end of discussion of revised manuscript (**lines 582-595**).

**Comment 6**: *Lines 288-290: Didn’t you measure aortic resistance at weeks 8 and 12?*

**Response:**

We measured aortic resistance at abdominal aorta proximal to Teflon strip coarctation or wrapping alone (sham) at different time frames. Figure 2D shows the changes of aortic resistance at pre-/post-coarctation and sacrifice in each group (sham, 4w, 8w and 12w).

**Comment 7**: *Figures: Please provide the sample size and the number of males/females per group in the figure legends.*

**Response:**

This recommendation is well taken. The sample size and the number of both genders in each group were added in figure legends of the revised manuscript.

**Comment 8**: *It is recommended to show the data as mean ± SD in all figures.*

**Response:**

This recommendation is well taken. The statistical data were shown as *mean ± SD* in all figures in the revised manuscript.

**Comment** 9: *It is recommended to include a limitation and perspective/significance section in the discussion.*

**Response:**

This recommendation is well taken. We added the section of limitation and perspective in the end of discussion (**lines 582-595**).

**Responses to comments of reviewer 2**

**Comment 1:** *The application of coarctation of the infrarenal abdominal aorta (AA) to induce hypertension and subsequent left ventricle (LV) hypertrophy in the current manuscript is questionable. The increase in arterial pressure and heart rate was only observed 12 weeks post-surgery (Fig 1 and Fig 2C), while all parameters assessing LV hypertrophy and myocardial fibrosis were not seen (Fig 5). In addition, the mean pressure in the aorta shows no difference pre- and post-surgery and at sacrifice (Fig 2A). These observations suggest the AA coarctation was either not sufficient or was not applied long enough to induce LV hypertrophy.*

**Response:**

This reviewer’s point is well taken. While hypertension and increased heart rate were detected in the cluster 1 pigs, no structural changes including cellular hypertrophy and interstitial fibrosis were detected. This phenomenon suggests that longer period is likely needed to detect structural changes in the heart. The lack of structural changes points to the nature of our observation being in the early stage of the hypertensive heart disease. We have added a paragraph to discuss the limitation and perspective of this study (**Lines 582-595**).

**Comment 2:** *Why are the diastolic BP and MBP in 8wk and 12wk groups before surgery (Fig 1 E&F) lower than other groups as measured by brachial cuff methods?*

**Response:**

Cuff method of the brachial arterial pressures, being convenient and non-invasive, is widely used. Unfortunately, this method is susceptible to interferences of various environmental factors, e.g. room temperature and body position, and hence can be highly variable. This is the reason we applied intra-arterial pressure (or ABP) method in the current study.

**Comment 3:** *The criteria used to separate clusters 1 and 2 are inappropriate. While cluster 2 consists of animals from all three treatment groups, 4 of 5 animals in cluster 1 are from the 12w group. Considering that the onset of hypertension was observed around 12 weeks post-surgery (Fig 1), the author should only classify and compare the cluster of animals from the same treatment group.*

**Response:**

Hypertension was detected in the carotid artery around 12 weeks post-surgery (Fig 1), but not in the abdominal aorta (Fig 2). Apparently, unknown factors in addition to the duration of AA coarctation may contribute to hypertension development. To address this issue, cluster analysis was applied. Cluster analysis is an unsupervised machine learning technique that performs its tasks without prior knowledge of the group definition. Such that, there are no predefined criteria to distinguish clusters 1 and 2. Besides, classifying and comparing groups of animals within the same treatment group (e.g. 12-week) is not statistically meaningful because of the insufficient number of animals.

**Comment 4:** *The mean pressure, hydraulic power, and hydraulic energy before surgery and at sacrifice were not different among all groups (Fig 2 A, E&F). Given the critique on the cluster classification above, the application of aortic hydraulic power and MAP&HR to predict HHD progression should be verified in a separate group of animals.*

**Response:**

We perform cluster analysis on all groups rather than within each group in order to find other independent factors in the treatment group. Besides, classifying and comparing groups of animals within the same treatment group (e.g. 12-week) is not statistically meaningful because of the insufficient number of animals.

**Comment 5:** *Why is the n in Figure 4 less than the one from Figure 3? Were carotid arterial pressure and heart rate only measured in selected animals in Figure 4? Or were the data of some animals excluded? Please provide justification if some data were excluded.*

**Response:**

For carotid ABP measurements, data from some pigs (n =7) were not used. In some cases, machine was not properly calibrated prior to experiments. In others, data were incomplete resulting from technical problems, including improper catheter placement due to loosening suture fixation and lodging the catheter tip to the arterial wall. We added the explanation in the revised manuscript (Method section, **lines 100-102**).

**Comment 6:** *Is the n of cluster 1 in Figure 5C equal to 2? If so, the authors should increase the number of samples in cluster 1 for LV hypertrophy assessment.*

**Response:**

We have added 2 samples in Figure 5C (n = 4) for accessing cellular hypertrophy of cardiomyocytes. To gain the most information from the experiments, we chose to perform cardiac myosin heavy chain immunofluorescence with nuclear counterstain. The immunofluorescence images were processed for two analyses: one for assessing the cross-sectional area, the other for nuclear density calculation. While processing these data, we detected errors made in previous processes. Therefore, we re-analyzed all data in Figures 5 and S1 and made changes in scale bars and values in the Y axis. The results were presented in the new version as Figures 5C (cross-sectional area) and 5D (nuclear density). The WGA immunostaining results (n = 2) were shown as S1 Fig.

**Comment 7:** Please add n of all groups in all figures.

**Response:**

We have added n values, including those of female and male sexes, in all of the figures.
