# Supplementary material for: Increased resting heart rate indicates high-workload hearts with augmented aortic hydraulic power in hypertensive pigs
Source: PLoS One. 2025 Jan 13;20(1):e0316607. doi: 10.1371/journal.pone.0316607 (PMC11729957; doi:10.1371/journal.pone.0316607)
Supplement: Attachment — Submitted filename: Response_to_Reviewers.docx [file pone.0316607.s006.docx]

**Responses to Reviewer**

**Response to comment of Reviewer ＃2**

**Comment:** *Lin and colleagues determined the pathophysiological changes of the heart in response to hypertension induced by infrarenal abdominal aorta coarctation in miniature pigs. The authors found that 12 weeks of moderate aortic coarctation increased systolic arterial pressure, mean arterial pressure, and heart rate. By clustering the animals into two groups based on the hydraulic power value in the aorta at sacrifice, the author reported that the group with increased aortic hydraulic power demonstrated hypertension and high heart rate compared to the other group and sham controls. Overall, this manuscript could be novel in understanding AA coarctation-induced hypertension as a potential model for studying the pathophysiology of hypertensive heart disease.*

*Comment to Response #3 to Reviewer 2: Please add this explanation of how cluster analysis is performed to line 289 of the revised manuscript. This information would solve the confusion of how these two clusters are classified.*

**Response:**

Thank you for your suggestion. To clearly convey the idea on cluster analysis, we changed the manuscript in two places, **lines 177-181** in Methods section and **lines** **293-295** in Results section.
